#  Systems Biology Profiling of AMD on the Basis of Gene Expression

**DOI:** 10.1155/2013/453934

**Published:** 2013-11-14

**Authors:** Mones S. Abu-Asab, Jose Salazar, Jingsheng Tuo, Chi-Chao Chan

**Affiliations:** Laboratory of Immunology, National Eye Institute, National Institutes of Health, Bethesda, MD 20892, USA

## Abstract

Genetic pathways underlying the initiation and progression of age-related macular degeneration (AMD) have not been yet sufficiently revealed, and the correlations of AMD's genotypes, phenotypes, and disease spectrum are still awaiting resolution. We are tackling both problems with systems biology phylogenetic parsimony analysis. Gene expression data (GSE29801: NCBI, Geo) of macular and extramacular specimens of the retinas and retinal pigment epithelium (RPE) choroid complexes representing dry AMD without geographic atrophy (GA), choroidal neovascularization (CNV), GA, as well as pre-AMD and subclinical pre-AMD were polarized against their respective normal specimens and then processed through the parsimony program MIX to produce phylogenetic cladograms. Gene lists from cladograms' nodes were processed in Genomatix GePS to reveal the affected signaling pathway networks. Cladograms exposed a highly heterogeneous transcriptomic profiles within all the conventional phenotypes. Moreover, clades and nodal synapomorphies did not support the classical AMD phenotypes as valid transcriptomal genotypes. Gene lists defined by cladogram nodes showed that the AMD-related deregulations occurring in the neural retina were different from those in RPE-choroidal tissue. Our analysis suggests a more complex transcriptional profile of the phenotypes than expected. Evaluation of the disease in much earlier stages is needed to elucidate the initial events of AMD.

## 1. Introduction

Age-related macular degeneration (AMD) is the main cause of permanent central blindness in the developed countries [1]. It manifests in drusen formation and degeneration/atrophy of the retinal pigmented epithelium (RPE) and neural retina, as well as the formation of abnormal choroidal capillaries [2, 3]. In addition to aging as the principal risk factor, there are others such as smoking, diet, and genetic predisposition [3, 4]. However, it is not yet sufficiently resolved the exact genetic pathways underlying the initiation and progression of AMD and the relationship between its genotypes and phenotypes [1].

Although a more recent clinical classification of AMD has been published recently [5], we are using that of Newman et al. [1] since the study specimens were categorized in the public data according to their phenotypes (see Table 1 for details), these encompass (1) dry AMD, (2) choroidal neovascularization (CNV) or Wet AMD, (3) geographic atrophy (GA) in macular region of RPE, (4) GA/CNV, (5) pre-AMD, and (6) subclinical pre-AMD. These phenotypes are typically the progressing manifestations of the disease, and their gene expressions may not harbor the early events responsible for the initiation and progression of the disease. A transcriptomic profiling of these phenotypes will elucidate the affected signaling pathways, reveal their similarities and differences, and clarify whether AMD's phenotypes represent a single disease or entities of an assemblage of diseases. In this study, we used systems biology analytical paradigm called parsimony phylogenetics to reveal the various transcriptomic profiles of AMD's subtypes. 

Further specific objectives of this analysis are to find out if gene expression profiling supports the current classification of phenotypes, to identify the shared gene expression aberrations among AMD's phenotypes, to find out if the transformations in the neural retina are similar to those in RPE-choroidal region, and to carry out class discovery in order to subtype AMD on the basis of gene expression profiles and answer whether it is a single disease or different disease entities.

To reach the above stated objectives, we have selected parsimony phylogenetics as the best systems biology tool to analyze microarray gene expression data of AMD obtained from public domains. Parsimony is an evolutionary analytical method that has been applied to mass spectrometry data of cancer [6], gene-expression of various diseases [7, 8], vaccine analysis [9], and systematics biology of taxa [10]. Parsimony algorithms are capable of utilizing shared derived gene expression aberrations to subtype specimens; they are very suitable for high dimensional heterogeneous data (i.e., with 10,000s of variables) [11]. 

## 2. Materials and Methods

Our analytical strategy can be summarized in the following steps: classify the patient specimens into clades (a cluster of specimens located on the cladogram) onto cladogram through parsimony analysis of their gene-expression data; identify shared genes with abnormal expression (termed synapomorphies in phylogenetic vocabulary) for each clade; and identify genetic pathways affected by abnormal gene expression for all AMD specimens and/or for each clade.

Dataset GSE29801 was downloaded from Geo Datasets of NCBI (http://www.ncbi.nlm.nih.gov/geo/query/acc.cgi?acc=GSE29801). The gene expression dataset of macular and extramacular encompassed specimens of retinas (55 normal, 13 pre-AMD, and 47 AMD) and retinal pigment epithelium (RPE-) choroid complexes (96 normal, 21 pre-AMD, and 60 AMD) [1]. The AMD specimens encompassed dry AMD without geographic atrophy (GA), choroidal neovascularization (CNV), and GA (Table 2). 

Pre-AMD and AMD gene expression values of retinal and RPE-choroidal specimens were polarized separately against their respective normal specimens (e.g., RPE-choroid data was polarized using normal RPE-choroid specimens data), and the new polarized data matrices were processed separately through MIX [12], a parsimony program of the PHYLIP package (http://evolution.genetics.washington.edu/phylip.html) to produce phylogenetic cladograms for both datasets (for details of this process see [7, 13]). The resulting cladograms were studied for meaningful interpretations and to fulfill the objectives stated in the introduction. Gene lists extracted from the cladograms nodes were processed in Genomatix GePS (http://www.genomatix.de/) to reveal the affected gene signaling pathway networks.

## 3. Results

For a more meaningful interpretation of the affected signaling pathways, our analysis focused on sampling different regions of the cladograms to reveal the diversity of the affected signaling pathways within AMD lesions. After the extraction of the synapomorphies at several locations of cladograms 1 and 2, we extrapolated from the synapomorphies the affected signaling pathways (Tables 3 and 4) by modeling the list of synapomorphies into Genomatix GePS. The sampled locations represented the basal, the middle, and upper sections of both cladograms. 

**Figure 1:**
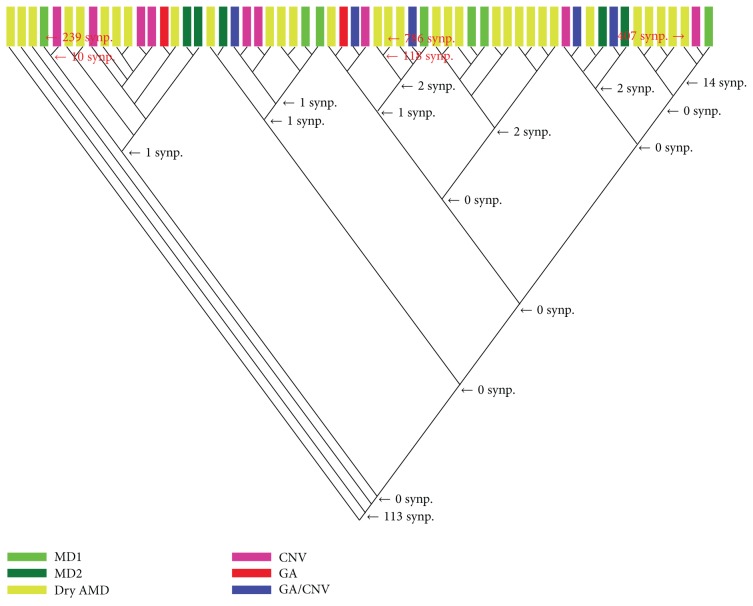
Cladogram of retinal specimens. The number of synapomorphies for major nodes is indicated to the right of the nodes, as well as for some specimens used as examples in the pathways analysis (numbers in red). Colors indicate AMD phenotypic subtypes.

**Figure 2:**
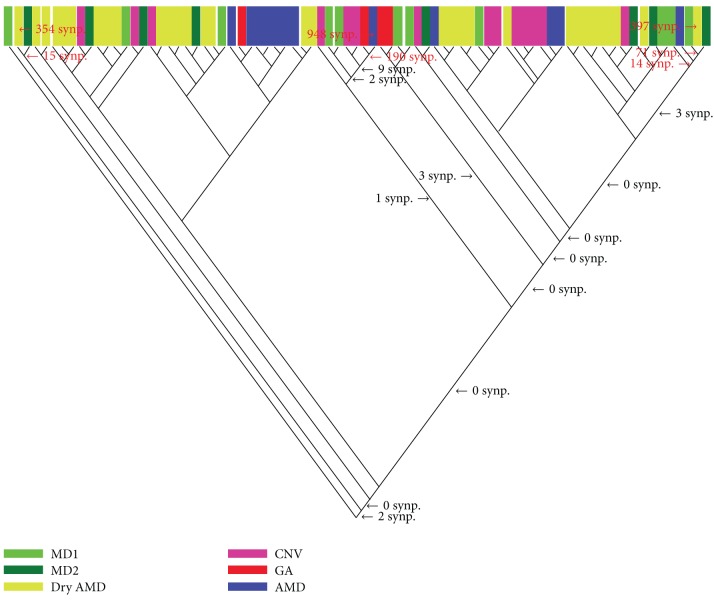
Cladogram of RPE-choroidal specimens. The number of synapomorphies for major nodes is indicated to the right of the nodes, as well as for some specimens used as examples in the pathways analysis (numbers in red). Colors indicate AMD phenotypic subtypes.

Each dataset analysis with MIX produced over 100 cladograms, and only one cladogram was selected (usually the first since the differences between the cladograms were in the upper minor branches) to represent each analysis (Figures 1 and 2). Interestingly, the analysis revealed the high heterogeneity of the specimens' gene expression irrespective of their phenotype in both retina and RPE-choroid complex. This was evident by the large number of cladograms produced (over 100) by the two datasets. Usually the fewer the number of cladograms produced the lower the heterogeneity and the higher the confidence in the results. Also supporting this conclusion were several aspects of the cladograms such as the terminal distribution of gene expression aberrations (see below). 

The specimens of each AMD phenotype did not cluster together to form a clade (a clade is a group of specimens sharing one or more abnormal gene expressions) but rather formed mixed clades that encompassed several phenotypes (Figures 1 and 2). Therefore, AMD phenotypes seemed not to be distinct entities according to their transcriptomic profiles of the retina or RPE-choroid complex suggesting that the clinically recognized phenotypes may not be supported by a classification based on gene expression abnormalities.

Macular and temporal extra-macular tissues of the same patient separated in most of the retinal and RPE-choroid complex sets but some clustered together (12–15%) indicating similar changes in both locations (macular and extramacular). This could be attributed to the diversity of the disease itself where it is similar in both locations in some patients and different in others, or could be due to sampling from similar locations.

The two cladograms (Figures 1 and 2) demonstrate that the AMD retina and RPE-choroid complex had slightly more transcriptomic subtypes than the currently recognized clinical phenotypes; for example, the number of clades within each cladogram is larger than the number of currently recognized phenotypes. 

Except for the majority of the retina AMD specimens (both macular and extramacular) that shared 113 synapomorphies (shared gene expression aberrations) most of the genetic aberrations were specimen-specific; however, there were a few synapomorphies defining a number of clades. Since AMD phenotypes did not form their respective clades, there were not any synapomorphies that defined any of the phenotype. While the retina clade was defined by 113 synapomorphies the RPE-choroid complex clade had only two synapomorphies; these are located at the basal section of the cladograms (Figures 1 and 2).

Tables 3 and 4 summarized the affected signaling pathways of the retina and RPE-choroid complex datasets respectively. Different signaling pathways were affected in the neural and nonneural tissues. Furthermore, the sampled sections of each cladogram had differently affected signaling pathways despite some minor overlap. While the changes in the retina were highlighted in apoptosis, cell cycle, cytoskeleton, and growth signaling pathway, those of the RPE-choroid complex showed affected signaling pathways of oxidative stress, inflammation, cell differentiation, and oncogenecity.

The samples of Table 4 were selected to represent the various locations of the cladogram of Figure 2 in order to explore the affected pathways among various clades. Some of the affected genes included C-X-C motif chemokine 12 (CXCL12) that is a chemokine strongly chemotactic for lymphocytes [14]; glial cell-derived neurotrophic factor (GDNF) that strongly promotes the survival of neurons [15] and prevents apoptosis of motor neurons; secreted frizzled-related protein 1 (SFRP1) that acts as a biphasic modulator of Wnt signaling, counteracting Wnt-induced effects at high concentrations and promoting them at lower concentrations [16]; which may also affect the differentiation of photo receptors [17]; and superoxide dismutase 1 (SOD1) that is associated with macular degeneration when its levels drops below normal [18]. More updates on other genes' functions can be obtained from http://www.ncbi.nlm.nih.gov/gene/. Unfortunately, since the cladograms of Figures 1 and 2 show that their clades do not have commonly shared aberrations along the axis of the cladograms, nothing can be said about directionality of gene change in AMD from these cladograms. The amount of heterogeneity in AMD advanced phenotypes seems to be vast and random.

## 4. Discussion

This study is the first transcriptomal analysis of the retina and RPE-choroid complex tissues from AMD patients and normal subjects by means of phylogenetic parsimony. The method is a data-based (not specimen-based) analytical paradigm that produces a hierarchical modeling of the specimens into clades (phylogenetic clusters) defined by their shared aberrations, which when identified reveal the affected signaling pathways. The parsimony cladogram is multidimensional tool that exposes the characteristics of its data. In this study, the large number of equally parsimonious cladograms that were produced from the two datasets displayed the massive heterogeneity of the expression pattern within or across the clinical classification of AMD. Each dataset produced over 100 cladograms, an unusually high number of cladograms for a dataset of anatomically-related specimens. However, such diversity in advanced degenerative disease could be expected since these diseases are a downhill path toward undifferentiation due to the deregulation of differentiation pathways, and their phenotypes can be reached through several ontogenic pathways. AMD follows the same pattern, and it should not be unexpected that its specimens have shown this considerable heterogeneity. 

However, it may be surprising to find that the transcriptional profiles of both datasets did not support the current classification of the AMDs phenotypes and that the neural retina is different from the RPE-choroid complex in their deregulated pathways. The clades produced by the parsimony algorithm did not even come close to the classification of Newman et al. [1] as evident in the cladograms of Figures 1 and 2. Further analyses of other data sets, such as metabolomic and proteomic data, are needed to confirm the findings. 

Pathological aberrations in general are usually divided into driver (clonal) and passenger (nonexpanded) [19]. On a cladogram, the driver aberrations are usually modeled at the basal nodes of the cladogram, while the passenger ones are at the terminal level of the clades or randomly distributed on the cladogram. In this study, the vast majority of aberrations are at the terminal level, that is, specimen-specific. This revelation that most of the gene expression aberrations are specimen-specific points out to two conclusions: the first is that the change is mostly patient-specific, and the second is that there are probably multiple etiologies for AMD. 

Our analysis is fundamentally different from that of Newman et al. who mainly used fold change (≥1.5) as their criteria to identify significantly expressed genes in AMD phenotypes. Ours differs in that we used the normal range of gene expression (minimum and maximum values of healthy specimens) as the cutoff for determining the under-and overexpressed genes per specimen. This was followed by a phylogenetic stratification of AMD retinal and RPE-choroid specimens to find the natural clusters (clades) and their affected pathways for each of the two groups of specimens. Since these two methods belong to two different schools of thought (specimen-based versus data-based), the congruence of their results was very weak. Therefore, gene lists and pathways of Newman et al. differed from ours. Furthermore, while Newman et al. claimed that their results supported the current phenotypic classification of AMD, we think that our unsupervised analysis did not support AMD's phenotypes [1]. Newman et al. maps of significant genes are the best indicators of gene expression heterogeneity within AMD's phenotypes and the difficulty in declaring any as global biomarkers; the vast majority of their claimed globally significant genes (Newman et al., Figure  2) are actually insignificant except for LOC100294179 in retina that is significant in dry AMD, GA, and CNV, and C10orf18 in RPE-choroid that is significant in CNV and MD. Our analysis indicated that the transcriptomal changes within the neural retina as a group of specimens were different from those in the RPE-choroid specimens, and these two sets of tissues differ from each other in their aberrations; therefore, it is most likely that there are no global biomarkers for AMD's phenotypes as defined in Table 1. This conclusion highlights the necessity of stratifying (subtyping) the disease as a priori to declare any aberrations as the global biomarkers of the disease subtypes [19]. As our analysis has shown here, there were different transcriptomal subtypes than the clinical ones.

AMD like all degenerative diseases can be bioinformatically modeled on a cladogram as a spectrum that ranges from early stages with initial events to advanced stages with later events. When specimens representing all stages of AMD are used to construct a cladogram, the ones harboring early stages of the disease will occupy the basal location of the cladogram while later stages follow. Therefore, revealing early events of AMD (i.e., gene expression deregulations that probably are not associated with morphological changes) requires the study of specimens that are less advanced in their pathology [19]. In this study, the identification of early events was not possible; this may be attributed to the lack of specimens with asymptomatic stages or relatively normal pathology of the disease. The presence of drusen in pre-AMD and subclinical specimens (see Table 1) may also represent part of an advanced stage of the disease rather than a pre-AMD or sub-clinical diagnosis since drusen may signify an advanced dysfunction of the mitochondria [20]. Although ophthalmologists rely on morphological criteria that appear to represent advanced events for AMD diagnosis, early detection of AMD transformations should be carried out on the basis of gene-expression profiling according to our analysis. Such early gene-expression profiles of AMD transformations have not yet been determined. Additionally, the subtyping of AMD may have to be delayed till early gene-expression profiles become available.

In spite of some slight overlap, the affected signaling pathways in AMD are different in the retina and RPE-choroid complex (Tables 3 and 4). In general, the retina specimens shared aberrations within apoptosis, cell cycle, cytoskeleton, and growth signaling pathways, and the RPE-choroid complexes showed aberrations related to inflammation, differentiation, hypoxia, and oncogenecity. It appears from the list of affected signaling pathways that the two tissue types are exposed to different stressors and therefore are responding in a different manner. Tables 3 and 4 detail the affected signaling pathways in the retina and RPE-choroid complex of AMD lesions.

In conclusion, AMD appears to be a diverse disease that involves two major independent but parallel pathological processes, one within the neural retina and the other within the RPE-choroid complex. In both areas, the transcriptomal changes are very heterogeneous and seem to be mostly patient-specific and involve various signaling pathways. Furthermore, the transcriptomal profiles seem to be incongruent with the clinical phenotypes, and the early gene expression events of AMD cannot be deciphered from the advanced phenotypes of the disease.

## Figures and Tables

**Table 1:** Description of AMD phenotypic subtypes according to Newman et al. [1]. Abbreviated names in the first column are used in labeling the cladograms' legends in Figures 1 and 2.

AMD phenotype	Alternative name	Description
MD1	Pre-AMD	Hard macular drusen (<63 *μ*m) only

MD2	Subclinical pre-AMD	Soft, distinct macular drusen (>63 *μ*m) Macular pigmentary irregularities without soft drusen

Dry AMD	Dry AMD (non-GA)	Soft, indistinct (>125 *μ*m) or reticular macular drusen Soft distinct macular drusen (>63 *μ*m) with pigmentary changes Soft indistinct macular drusen with pigmentary changes

GA	Geographic atrophy	Sharply demarcated area of apparent absence of the RPE (>175 *μ*m) involving central macular region

CNV	Wet AMD	Subretinal choroidal neovascularization

GA/CNV		Geographic atrophy with choroidal neovascularization

**Table 2:** The study collection's clinical phenotypes and the number of their specimens. Data source: GSE29801 at Geo Datasets of NCBI (http://www.ncbi.nlm.nih.gov/geo/query/acc.cgi?acc=GSE29801).

Dx	Retina	
	Macular	Extramacular
Normal (*n* = 55)	28	27

Pre-AMD (*n* = 13)	MD1 = 4	MD1 = 4
	MD2 = 3	MD2 = 2

AMD (*n* = 47)	Dry = 15	Dry = 16
	CNV = 5	CNV = 4
	GA = 1	GA = 1
	GA/CNV = 3	GA/CNV = 2

	RPE-choroid	

Normal (*n* = 96)	48	48

Pre-AMD (*n* = 21)	MD1 = 6	MD1 = 5
	MD2 = 4	MD2 = 4

AMD (*n* = 60)	Dry = 15	Dry = 15
	CNV = 5	CNV = 5
	GA = 2	GA = 2
	GA/CNV = 2	GA/CNV = 2
	Undetermined = 6	Undetermined = 6

**Table 3:** Affected retinal signaling pathways at different locations of cladogram in Figure 1. Sample identification follows http://www.ncbi.nlm.nih.gov/geo/query/acc.cgi?acc=GSE29801.

First node Shared by all retinal specimens	RetMD1-106 (Sample GSM738713) Lower part of the cladogram	Specimen: RetDRY98 (Sample GSM738705) Middle part of the cladogram	Specimen: RetDRY70 (Sample GSM738677) Upper part of the cladogram
(1) Apoptosis (2) Cell cycle (3) Cytoskeleton (4) Differentiation (5) Growth (6) Insulin metabolism	(1) Apoptosis (2) Cell cycle (3) Development (4) Growth (5) Neurotransmission (6) Transcription activation (7) Tumor suppression	(1) Cytokine receptor degradation signaling (2) Cytosolic calcium ion concentration elevation (through IP3 receptor) (GPCR signaling (G alpha q)) (3) EGFR1 (4) ERK cascade GPCR signaling (G alpha s, PKA, and ERK) (5) Protein binding (6) Proteolysis	(1) Amyloid metabolism (2) Apoptosis (3) Cell cycle (4) Cytoskeleton (5) Immunoregulation (6) Inflammation (7) Lipid metabolism (8) Retinoid metabolism (9) Ribosomal proteins (10) Telomere metabolism

**Table 4:** Affected RPE-choroidal signaling pathways at different locations of cladogram in Figure 2. Sample identification follows http://www.ncbi.nlm.nih.gov/geo/query/acc.cgi?acc=GSE29801. Updates on genes' functions can be obtained from http://www.ncbi.nlm.nih.gov/gene/.

Dry 135 (Sample GSM738566) Lower part of the cladogram	Dry 145 (Sample GSM738575) Middle part of the cladogram	Dry 136 (Sample GSM738567) Upper part of the cladogram
(1) CXCL12: activates lymphocytes (2) GDNF: promotes the survival and differentiation of dopaminergic neurons (3) MAPK1: proliferation, differentiation, transcription regulation, and development (4) PIK3CA: oncogenic (5) SFRP1: soluble modulator of Wnt signaling (6) SOD1 superoxide dismutase 1	(1) ABL1 protooncogene implicated in cell differentiation, division, adhesion, and stress response (2) CAV1: cell cycle (3) CCL20: inflammation (4) CREB1: a transcription factor, cAMP pathway (5) CRY2: insulin metabolism (6) ERCC1: DNA repair (7) ESR1: hormone binding, DNA binding, and activation of transcription (8) IL8: inflammatory response (9) INS: insulin (10) MSN: cytoskeleton (11) MT1A: cytoskeleton, and so forth (12) PML: tumor suppressor (13) SERPINE1: inhibitor of fibrinolysis (14) TBP: assembly of transcription complex, and acts as a channel for regulatory signals (15) TMSB4X: cytoskeleton, proliferation, migration, and differentiation	(1) CAV1: cell cycle (2) CCL5: inflammation (3) CXCL12: activates lymphocytes (4) EGF: growth, proliferation, and differentiation (5) PPARA peroxisome proliferator-activated receptor alpha
